# Circulating and Tumor-Infiltrating Myeloid-Derived Suppressor Cells in Patients with Colorectal Carcinoma

**DOI:** 10.1371/journal.pone.0057114

**Published:** 2013-02-20

**Authors:** Bin Zhang, Zhijun Wang, Liangliang Wu, Meng Zhang, Wei Li, Jianhua Ding, Jun Zhu, Huafeng Wei, Ke Zhao

**Affiliations:** 1 Department of Colo-Rectal Disease Surgery, The Second Artillery General Hospital, Beijing, China; 2 Department of Interventional Radiology,PLA General Hospital, Beijing, China; 3 Cancer Center Lab, PLA General Hospital, Beijing, China; 4 Department of Geriatric Neurology, Brain Hospital Affiliated to Nanjing Medical University, Nanjing, China; 5 International Joint Cancer Institute, Second Military Medical University, Shanghai, China; New York University, United States of America

## Abstract

Myeloid-derived suppressor cells (MDSCs) are a heterogeneous family of myeloid cells that suppress T cell immunity in tumor-bearing hosts. In patients with colon cancer, MDSCs have recently been described as Lin^−/low^HLA-DR^−^CD11b^+^CD33^+^ cells correlating with cancer stage, metastasis and chemotherapy response. To learn in more detail the dynamic change and clinical relevance of circulating and tumor-infiltrating Lin^−/low^HLA-DR^−^CD11b^+^CD33^+^ MDSC in colorectal cancer, we harvested the blood from 64 patients with varying stage of colorectal cancer and tumor and matched paraneoplastic tissues from 5 patients with advanced colorectal cancer, subjected them to multicolor flow cytometric analysis of percentage, absolute number and phenotype of MDSC and finally characterized their immunosuppressive functions. Our results demonstrate that peripheral blood from colorectal cancer patients contains markedly increased percentage and absolute number of Lin^−/low^HLA-DR^−^CD11b^+^CD33^+^ MDSCs compared with healthy individuals, and this increase is closely correlated with clinical cancer stage and tumor metastasis but not primary tumor size and serum concentrations of cancer biomarker. A similar increase of MDSCs was also observed in the tumor tissues. Phenotyping MDSCs shows that they express high CD13 and CD39, low CD115, CD117, CD124 and PD-L1, and devoid of CD14, CD15 and CD66b, reminiscent of precursor myeloid cells. MDSCs from cancer patients but not healthy donors have the immunosuppressive activity and were able to inhibit *in vitro* autologous T-cell proliferation. Collectively, this study substantiates the presence of increased immunosuppressive circulating and tumor-resident Lin^−/low^HLA-DR^−^CD11b^+^CD33^+^ MDSCs in patients with colorectal cancers correlating with cancer stage and metastasis, and suggests that pharmacologic blockade of MDSCs should be considered in future clinical trials.

## Introduction

Human colorectal cancer is the third most common cancer and the fourth leading cause of cancer-related deaths worldwide 1]. The tumorigenesis of colorectal cancer involves numerous pathological factors and transformation of multiple genes 2,3]. It has been shown that chronic mucosal inflammation is associated with the development of colorectal cancer 4,5]. Like most solid cancers, colorectal cancer exhibits immune/inflammatory infiltrates with upregulation of characteristic ‘inflammatory signature’ genes 4,5]. Although infiltrating CD4^+^ Th1 cells and CD8^+^ cytotoxic T cells sign a positive prognosis in colorectal cancer 6–8], the immunosuppressive regulatory T cells and myeloid cells promote tumorigenesis 4,5]; therefore, characterization of these immunosuppressive cells has an important implication for diagnosis and therapeutics of this cancer.

Myeloid-derived suppressor cells (MDSCs) are a heterogeneous population composing of cells at several stages of differentiation of the myeloid lineage (Lin), accumulate in the blood, lymph nodes, bone marrow, and tumor sites in patients and experimental animals with cancer, and are capable of inhibiting both innate and adaptive immune responses 9,10]. Recent research has documented that expansion and accumulation of MDSCs constitute one of the important mechanisms of tumor immune evasion 11,12]. The increased presence of circulating inflammatory myeloid cells in both peripheral blood and tumor tissue influences tumor progression through local immune suppression and stimulation of tumor neovasculogenesis 13,14]. MDSCs have been shown to express different surface markers, depending both on the stage of myeloid development examined and the differentiation context provided by factors secreted by cancer cells 15]. In mice, MDSCs were described as CD11b/Gr-1-double-positive cells 16]. Furthermore, different subsets of murine MDSCs recently have been identified based on the expression of the Gr-1 antigens Ly-6G (granulocytic MDSCs) and Ly-6C (monocytic MDSCs) 16]. It is now hypothesized that murine MDSCs originate in the bone marrow of tumor-bearing mice, accumulate in the periphery and circulation as the tumor progresses and finally enter the malignant tissue, where they become activated and subsequently acquire immunoregulatory and immunosuppressive properties after exposure to local tumor-derived factors 9,10,17].

Unlike mouse MDSCs, the human counterpart does not have a universal marker and their function and pathophysiological relevance of putative MDSCs in human oncology is less well defined 9,18,19]. Analogous to the situation in murine MDSC, it is possible to broadly classify the MDSC phenotypes described in the literature as being predominantly granulocytic (expressing markers such as CD15, CD66b, CD33) or monocytic (expressing CD14). The frequency of each MDSCs subset appears to be influenced by cancer type. Patients with renal cancer have immunosuppressive CD14^−^CD15^+^CD11b^+^CD66b^+^ granulocytic MDSCs 20], whereas CD14^+^HLA-DR^−/low^ monocytic MDSCs circulate in the blood of patients with melanoma, multiple myeloma, prostate cancer, hepatocellular carcinoma or head and neck cancer 21–24]. More recently, it has been reported that the suppressive activity of human MDSCs resides in a CD14^+^S100A9^+^ inflammatory monocytes in non-small cell lung cancer (NSCLC) patients 25]. In addition, a population with Lin^−/low^HLA-DR^−^CD11b^+^CD33^+^ phenotype has been demonstrated to have the features and properties of MDSCs in the blood from patients with glioblastoma, breast cancer, colon cancer, lung cancer or kidney cancer 15,26–29]. These cells do not express either monocytic (CD14) or granulocytic (CD15, CD16) markers, and therefore cannot be categorized into one of the two main populations described above. Examination of nuclear morphology of these cells found that they have predominantly a precursor phenotype. The frequency and number of these cells has been shown to reflect the tumor burden, and a high frequency correlates with a poor prognosis and radiographic progression in a small number of patient with breast or colorectal cancer 27].

To more detailed learn the dynamic change and clinical relevance of circulating and tumor-infiltrating Lin^−/low^HLA-DR^−^CD11b^+^CD33^+^ MDSC in the colorectal cancer, in this study, we harvested the blood from 64 patients with varying stage of colorectal cancer and tumor and matched paraneoplastic tissues from 5 patients with stage III colorectal cancer, subjected them to multicolor flow cytmetric analysis of percentage, absolute number and phenotype of MDSC, and finally characterized their immunosuppressive functions. Our findings might have an important implication for the treatment of colorectal cancer.

## Materials and Methods

### Ethical statement

All patients and healthy donors provided written informed consent prior to blood sampling and/or tumor tissue harvesting. The research protocol was approved by the Institutional Review Board of Second Artillary General Hospital and Second Military Medical University.

### Patients

Patients and healthy donors blood samples were collected from healthy volunteers (N = 32) and patients with colorectal cancer (N = 64) who were seen at the Department of Institution of Hepatobililary and Gastrointestinal Diseases, Second Artillary General Hospital, Beijing. All patients were diagnosed with colorectal cancer for the first time, and had not been previously treated. Table 1 shows the clinical characteristics of all patients in this study.

**Table 1:** Patient characteristics.

	CRC	Limited^a^	Metastatic^a^
		(UICC I+II)	(UICC III+IV)
Characteristics	(n = 64)	(n = 11)	(n = 53)
Age^b^ (years)	64.7±12.8	68.3±12.5	63.9±12.8
Gender			
Male	28	4	24
Female	36	7	29
TNM stage^c^			
I	6	6	
II	5	5	
III	15		15
IV	38		38
Tumor site^d^			
Poximal	18	3	15
Distal	46	8	38
Histologic grade			
Well/moderate	51	9	42
Poor/undifferentiated	13	2	11

a Colorectal cancer with limited disease corresponds to stage I and II, and those with metastatic disease corresponds to stages III and IV.

b Mean ± standard deviation.

c Stage according to the TNM classification for Colorectal cancer (UICC).

d Tumor site was classified as proximal to and including, or distal, to the splenic flexure.

### Antibodies and flow cytometric analysis

All monoclonal antibodies used in the study were purchased from Biolegend. For identification of circulating MDSCs, we used PE-Cy7-anti-HLA-DR, PE-anti-CD11b, APC-anti-CD33 and FITC-anti-Lineage antibodies. For analysis of tumor-infiltrating MDSCs, we used FITC-anti-CD45, PE-anti-CD11b and APC-anti-CD33 antibodies. For phenotyping circulating MDSCs, we first gated MDSCs using anti-HLA-DR/Lineage/CD33 antibodies and then costained with PE-anti-CD13, CD34, CD-39, CD-66b, CD73, CD115 (CSF-1R), CD124 (IL-4Rα), PD-1 (CD279), PD-L1 (CD274), and PD-L2 (CD273). Isotype-matched antibodies were used as controls. Staining was performed on fresh venous blood collected in EDTA-coated vacutainer tubes (BD Biosciences) or tumor-infiltrating immune cells isolated described below. Briely, 100 µL of blood or 106 tumor-infiltrating cells was mixed with 1 µL of each antibody on a 96-well plate. Plates were incubated at room temperature for 30 min. After incubation, each blood sample was mixed with 2 mL of ACK lysis buffer and incubated for 10 min. Samples were washed with FACS buffer (2% BSA in PBS, 0.09% sodium azide). Pellets were resuspended in 300 µL of FACS buffer. Samples were acquired on a Cytomics FC 500 MPL (Beckmam Coulter) and analyzed by FlowJo software (TreeStar, Inc.). The absolute number of MDSCs was calculated as follows: [total white blood cell count (cells/ µL)×percent MDSCs]/100 or [total tumor-infiltrating immune cell count (cells/100 mg tumor)×percent MDSCs]/100.

### Cell isolation

Peripheral blood mononuclear cells (PBMCs) were isolated from the peripheral blood of healthy donors and cancer patients by density gradient centrifugation. In brief, blood was collected in EDTA-treated tubes, diluted 1/2 with RPMI 1640 medium, and carefully layered onto a density gradient Ficoll-Hypaque (GE Healthcare). After centrifugation, the band of PBMC was aspirated; PBMC were washed three times with ice-cold PBS containing 1% of human serum. Cell viability was checked by trypan blue dye exclusion.

For isolation of tumor-infiltrating immune cells, freshly resected tumor and matched paraneoplastic tissues from patients with stage III colorectal cancer (100 mg) were cut into pieces and then enzymatically digested in RPMI 1640 medium containing 500 µg/ml liberase and 200 µg/ml DNase (Roche) for 45 min at 37°C with gentle shake every 10 min until all the tumor tissue had resolved into a cell suspension. The resulting cell suspension was filtered through 70 µL cell strainers (BD Biosciences) and subjected to the density centrifugation as described above.

### In vitro suppression assay

PBMC were isolated from 20 ml of whole blood taken from one healthy donor and two patients with stage IV colorectal cancer, and then costained with anti- HLA-DR/CD33/CD3 antibodies. HLA-DR^−^CD33^+^ MDSCs and CD3^+^ T cells were purified by MoFlo^TM^ XDP cell sorting system (Beckmam Coulter). The purity of all isolated cell populations was >95% (data not shown). For functional analysis, purified T cells were labeled with 2 µM CFSE (Invitrogen Molecular Probe) according to the manufacturer's instructions, and consequent CFSE-labeled T cells (1×10^5^) were cocultured at 2∶1 ratios with HLA-DR^−^CD33^+^ MDSCs in the absence or presence of soluble anti-CD3 (2 µg/mL) and anti-CD28 (0.5 µg/mL) antibody. After 72 hours, proliferation was measured by CFSE dilution as described previously 15].

### Statistical analysis

Statistical analysis was performed on GraphPad Prism 5.0 software (GraphPad Software, United States). Unpaired Student's *t* test (Mann-Whitney test) and unparametric Spearman test were used to assess the differences and correlation between the study groups respectively. P value <0.05 was considered statistically significant.

## Results

### The percentage and absolute number of Lin^−/low^HLA-DR^−^CD11b^+^CD33^+^ MDSCs were elevated in patients with colorectal cancer

**Figure 1:**
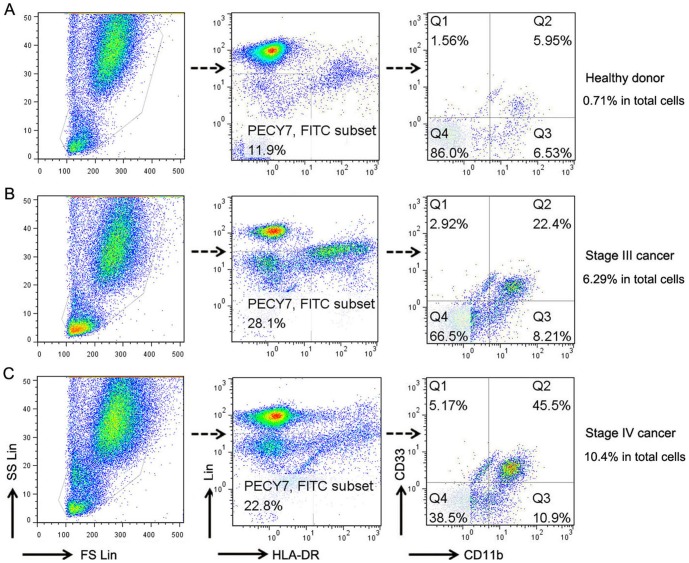
MDSCs definition by flow cytometry. To determine the percentage of MDSCs in patients, fresh whole blood was incubated with a combined anti-Lin, HLA DR, CD33 and CD11b monoclonal antibodies. Acquired cells were first gated (PECY7/FITC subset) based on the expression of Lin and HLA DR. PECY7/FITC subset was comprised of Lin^−/low^ and HLA DR^−^ cells. Within this population the fraction of cells expressing both CD33 and CD11b was determined. Therefore, MDSCs were defined as Lin^−/low^, HLA DR^−^, CD33^+^ and CD11b^+^ cells. MDSCs percentage was calculated as percentage of total nucleated cells in whole blood samples. Representative flow cytometric dotplots of a healthy volunteer (A), a patient with stage III (B) and IV (C) colorectal cancer.

**Figure 2:**
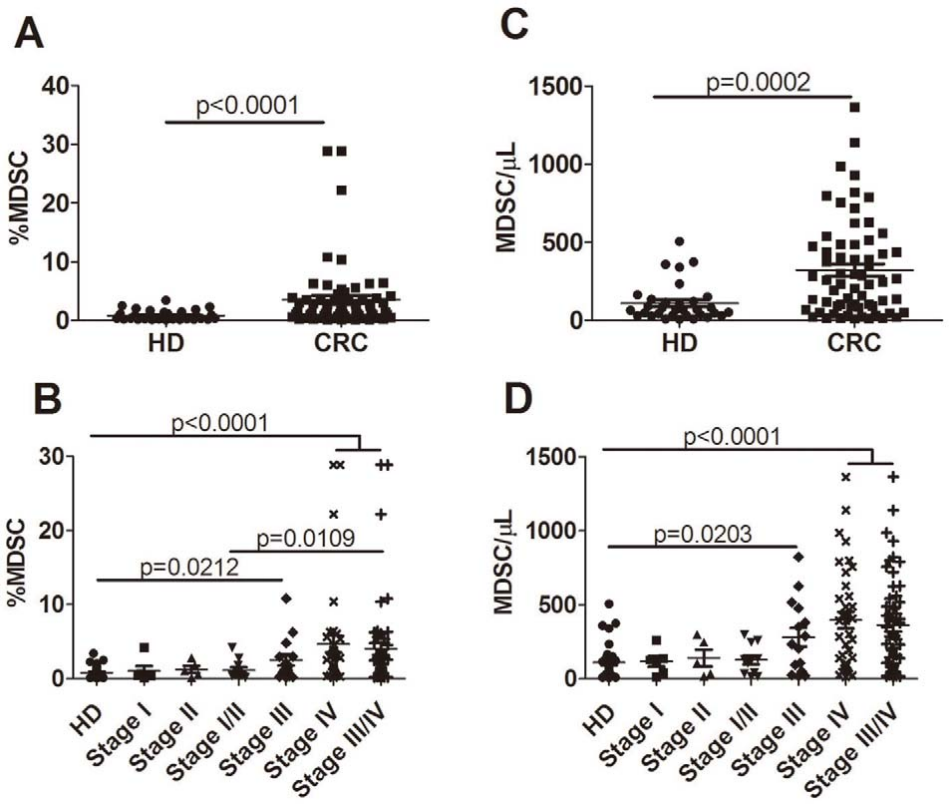
Significantly elevated levels of circulating MDSCs in patients with advanced colorectal cancer. Whole blood drawn from patients prior to any therapy was analyzed for the presence of MDSCs. Results showed a significantly higher percentage (A) and absolute number (C) of circulating MDSCs in cancer patients (n = 64) versus normal volunteers (n = 32). Moreover, both greater percentages (B) and absolute numbers (MDSCs/ µL) (D) of MDSCs were only detected in cancer patients with stage III/IV disease relative to patients with clinical stages I/II tumors.

Previous studies have described significantly elevated levels of circulating Lin^−/low^HLA-DR^−^CD11b^+^CD33^+^ MDSCs in the peripheral blood of patients with advanced cancer of multiple types, including colon cancer 27]; therefore in our study, we analyzed the presence and dynamic evolution of Lin^−/low^HLA-DR^−^CD11b^+^CD33^+^ MDSCs in the freshly obtained whole blood from colorectal cancer with varying stage (Table 1 for demographics). Percentages of MDSCs were calculated as a percentage of total cells. Representative flow cytometric data of a normal healthy donor (HD), two patients with stage III and IV colorectal cancer are shown in Fig. 1. Consistent with previous study, a significantly higher level of circulating MDSCs in cancer patients were observed compared with normal volunteers (mean 0.818% vs 3.554%; p<0.0001) (Fig. 2A).

We then divided patients by clinical cancer stage. Significant differences were seen for the percentage of circulating MDSCs between healthy donors and patients with stages III/IV solid tumors (mean 0.818%vs2.501%, 4.667% and 4.054% for HD vs stage III, IV and III/IV; p = 0.0212, p<0.0001, p<0.0001 respectively) (Fig. 2B). The patients with stage I/II cancer contained the mildly increased percentage of MDSCs which did not reach the statistical significance (mean 0.8180% vs 1.063%, 1.245% and 1.146% for HD vs stage I, II and I/II; p = 0.7037, p = 0.4111, p = 0.4276 respectively). Compared with those stages I/II tumor, advanced stages III/IV cancer had significantly higher percentage of circulating MDSCs (1.146% vs 4.054% p = 0.0109).

Further analysis of absolute number of MDSCs revealed the similar change. As shown in Fig. 2C and D, there was a significantly increased number of MDSCs in cancer patients relative to normal volunteers (mean 110.6 vs 322.4 cells/ µL; p = 0.0002) with stage III/IV cancer having much higher numbers compared with HD or stage I/II cancer (mean 117.1, 140.2, 280.0 and 395.6 cells/ µL for stage I, II, III and IV; p = 0.4961, p = 0.6730, p = 0.0293 and p<0.0001 respectively compared with HD) although considerable heterogeneity were present within patients with stage III/IV cancer, which was concordant with previous observation.

**Figure 3:**
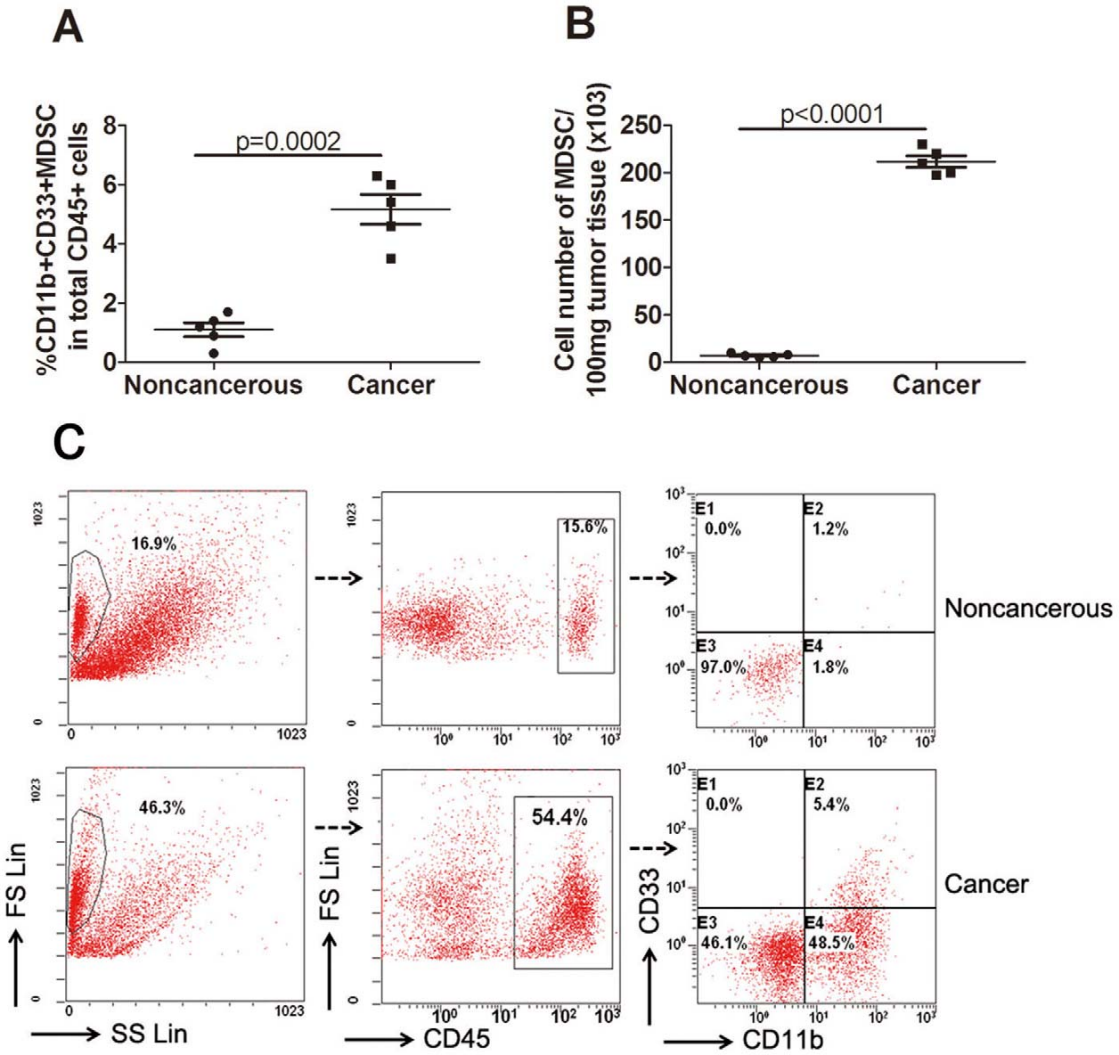
Significantly elevated levels of tumor-infiltrating MDSCs in patients with advanced colorectal cancer. Tumor tissues and matched surrounding non-cancerous tissues were obtained from 5 patients with stage III colorectal cancer. Tissues were disaggregated as specified in Materials and Methods and stained with fluorochome-labeled antibodies against CD45, CD11b and CD33. Results showed a significantly higher percentage (A) and absolute number (B) of tumor-infiltrating MDSCs compared with matched non-cancerous tissues. Representative dotplots of MDSCs from a tumor tissue and matched non-cancerous tissue.

We next examined the colorectal tumor tissue infiltrate for presence of myeloid cells. Tumor cell suspensions were prepared from fresh surgically excised colorectal tumor tissues of five patients with stage III colorectal cancer and stained with fluorochrome-conjugated antibodies against markers of myeloid cells. As shown in the Fig. 3A, compared with the matched paraneoplastic tissue, the percentage of MDSCs in the total CD45^+^ tumor-infiltrating leukocytes were markedly increased in the tumor tissue (mean 1.10% vs 5.16%; p = 0.0002). The difference in the absolute number of these cells was even more prominent (Fig. 3B; mean 7.200 vs 211.6×103 cells/100 mg tumor tisse; p<0.0001). The representative flow cytometric dotplots were shown in Fig. 3C.

### The circulating MDSCs correlated with the tumor metastasis

**Figure 4:**
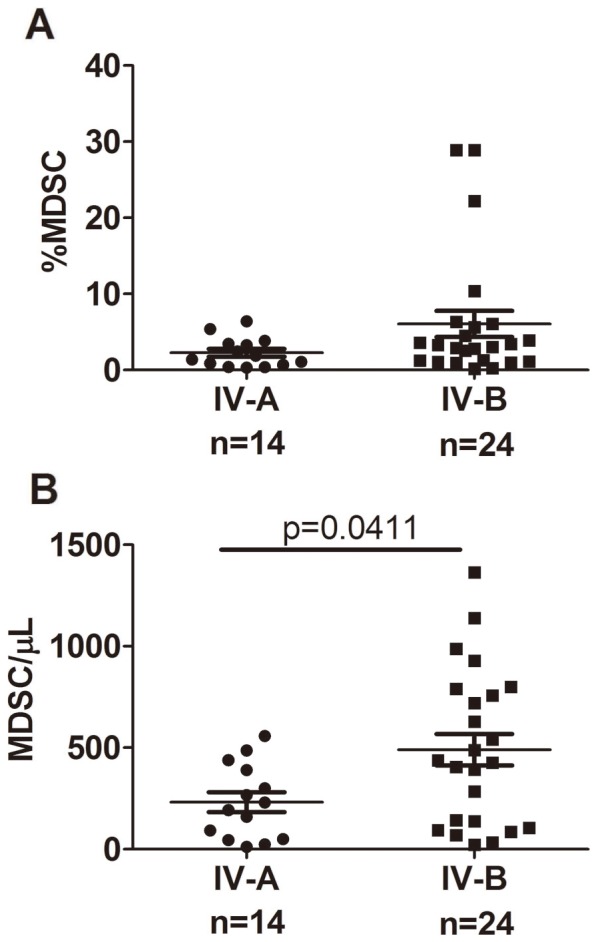
Circulating MDSCs levels correlate with extensive metastasis in patients with stage IV colorectal cancer. Patients with IV stage cancer were divided into IV-A stage (metastasis confined to one organ or sites; n = 14) and IV-B stage (metastases in more than one organ/sites or the peritoneum; n = 24) cancer. Patients with extensive metastasis had markedly increased percentages (A) and significantly higher (B) of absolute numbers of circulating MDSCs.

Previous studies in murine tumor models have reported that number of MDSCs is closely related with the tumor burden with the higher level of MDSCs in spleen from mice with larger tumor. To address the relationship between MDSCs accumulation and tumor burden in colorectal cancer, we divided advanced tumor into stage IV-A and stage IV-B tumor with limited and extensive metastasis respectively. As shown in Fig. 4A, the patients with extensive metastasis had significantly more absolute number of circulating MDSCs (mean 232.3 vs 490.9 cells/ µL; p = 0.0411); although the percentage of MDSCs from the same patients markedly increased (Fig. 4B), it did not reach statistical significance due to the big variation in these samples (mean 2.277% vs 6.061%; p = 0.1342).

In addition, we also analyzed the correlation between the MDSCs and primary tumor size and serum concentration of cancer biomarker tested pre-operatively. Of 64 cases analyzed, 18 cases had the record of primary tumor size measured when tumors were dissected. No correlation was observed between the percentage and absolute number of MDSCs and primary tumor size (Figure S1). Nor was correlation present between the serum concentration of cancer biomarker CEA and CA199 and MDSCs accumulation (Figure S2).

### Phenotypic analysis of Lin-HLA-DR-CD11b+CD33+ MDSCs

**Figure 5:**
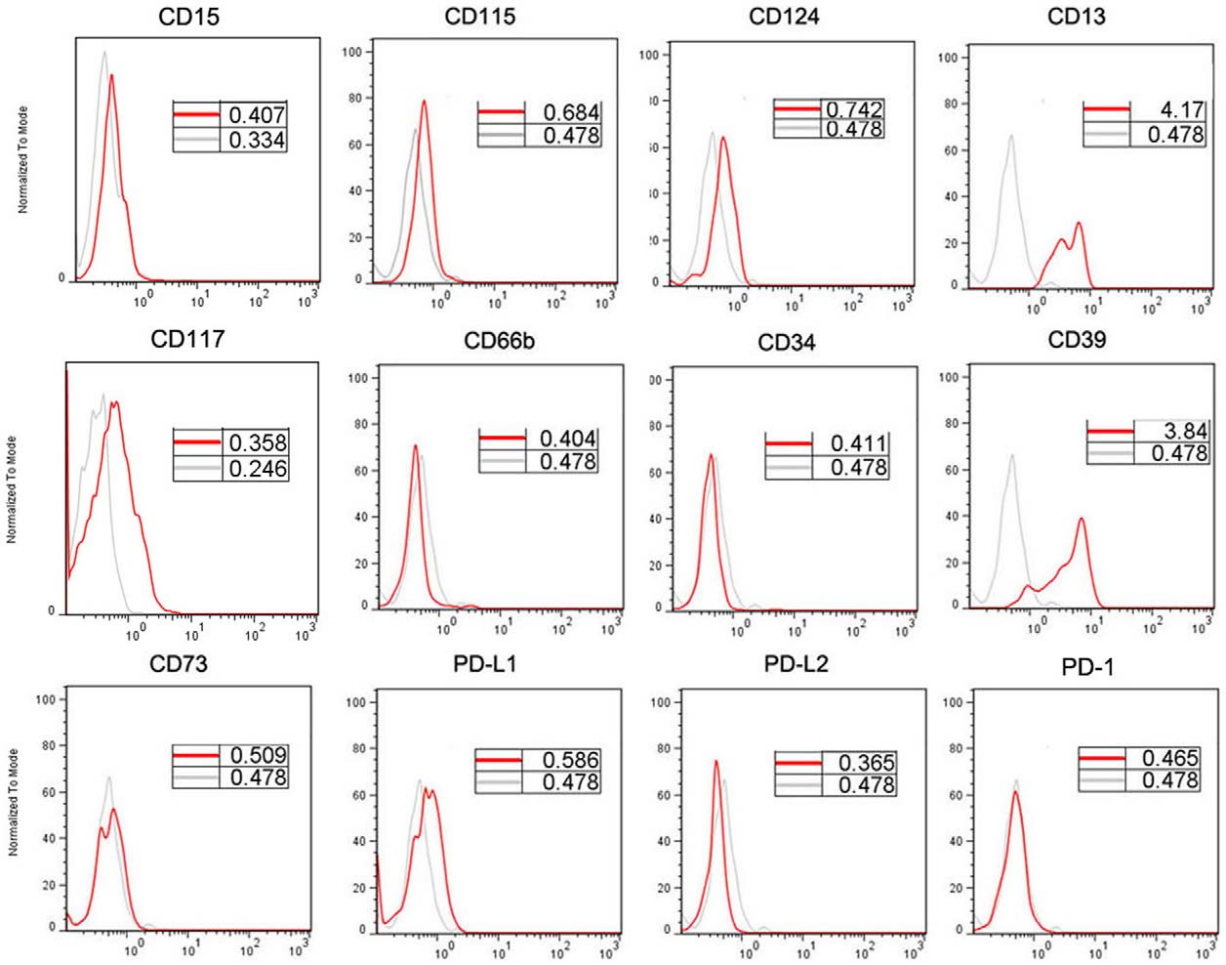
Phenotypic analyses of Lin^−/low^HLA-DR^−^CD11b^+^CD33^+^ MDSCs in colorectal carcer patients. The expression of putative MDSCs markers, markers of mature and immature myeloid cells, and markers associated with immune regulation was evaluated relative to isotype control (gray histograms).

To further characterize circulating MDSCs in colorectal cancer patients, we measured the expression of specific myeloid and lymphoid markers on these MDSCs using multicolor flow cytometry. Results from a representative experiment are shown in Fig. 5. Circulating MDSCs from colorectal cancer patients express high CD13, low CD115, CD117 and CD124 (IL-4Ra) and negative CD14, CD15, CD66b and CD34. Intriguingly, they also express high level of CD39 but not CD73, two ecto-nucleotidases involving degradation of immunstimualtory ATP into immunosuppressive adenosine. We also did not detect the expression of intracellular CD73 (data not shown). In addition, we found that MDSCs from colorectal cancer patients express low levels of PD-L1 but not PD-L2 and their receptor PD-1, similar to the mouse MDSC 16]. We did not observe the clear difference of expression of these markers in MDSCs from patients or healthy donors (data not shown).

### Immunosuppressive effect of tumor-derived MDSCs

**Figure 6:**
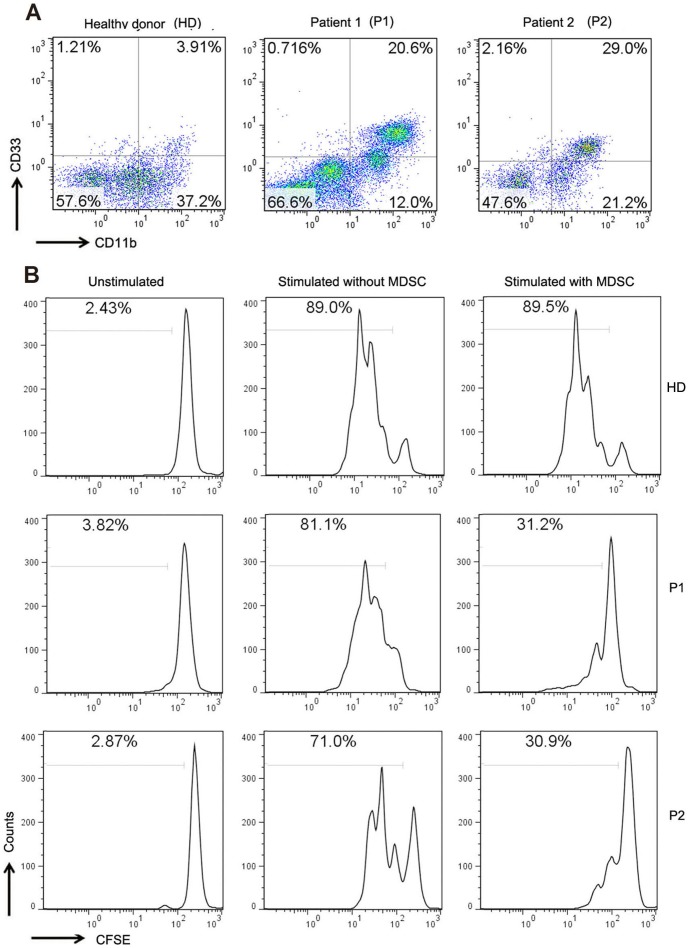
Isolated myeloid cells (HLA-DR^−^CD33^+^) from cancer patients inhibits CD3^+^ T-cell proliferation. MDSCs (HLA-DR^−^CD33^+^) and CD3^+^ T cells were isolated from freshly drawn blood from one normal volunteers (HD), two patients with stage IV cancer (P1 and P2). Percentage of circulating MDSCs from these samples (A). Purified CD3^+^ T cells were labeled with CFSE and cultured with or without MDSCs at a ratio of 2∶1 in the absence or presence of anti-CD3/CD28 antibody. After 3 days, cells were harvested and analyzed for CFSE dilition. Graph shows CFSE^+^CD3^+^ T cells and the percentage of the cells in division included.

To determine if MDSCs inhibit the activity of T cells, we purified MDSCs from one healthy donor and two patients with stage IV cancer (Fig. 6A) and cocultured them with or without CFSE-labeled autologous CD3^+^ T cells at the 1∶2 ratios in the absence or presence of CD3/CD28 antibody stimulation for 3 days. As shown in Fig. 6B, addition of MDSCs from cancer patients markedly suppressed the division of autologous T cells (62% and 56% inhibition for patient 1 and 2); however, no inhibitory effect was observed for MDSCs from healthy donor. The result indicates that tumor-derived MDSCs but not MDSCs from healthy donor have the immunosuppressive effect on T cells.

## Discussion

In view of the importance of MDSCs as one of critical players in mediating cancer immune evasion, consistently identifying and characterizing these cells is urgently needed. Previous work established the presence of the Lin^−/low^HLA-DR^−^CD11b^+^CD33^+^ circulating MDSCs in the blood from colon cancer patients that suppressed T cell proliferation and cytokine production 27]. In this study, we extent the previous findings by analyzing percentage, absolute number, phenotype and immunosuppressive function of circulating and tumor-infiltrating MDSCs from a cohort of colorectal cancer sample with varying stage and correlating MDSCs with the clinical parameters.

Since the different sample processing methods can alter the measurement of MDSCs by flow cytometry 30], to be consistent and comparative with prior study, we used whole blood to analyze the percentage and absolute number of circulating MDSCs, which was consider to be a more accurate method for enumeration of MDSCs in patient samples 27]. Concordant with previous reports, we found that MDSCs levels in cancer patients are significantly higher when compared to healthy donors 15,27,31]; however, distinct with previous study of colon cancer 27], the significant difference was only observed between patients with advanced tumor and healthy donors while patients with stage I/II cancer had a moderately increased percentage and absolute number of circulating MDSCs. The slight discrepancy regarding the MDSCs levels in patients with stage I/II cancer may be at least explained by the cancer type of sample since patient population (106 cases) in the previous study included a diverse group of patients with solid tumors of multiple type, including 10 cases of colon cancer with unspecified stage 27]. As they pointed out, it is quite possible that MDSCs production may be tumor specific. In addition, we only enrolled 11 cases with stage I/II colorectal cancer, and it is very likely that we may observe the significant increased MDSCs levels if more patients with early stage colorectal cancer would be included. A recent study shows the significantly increased percentage of circulating and tumor-resident HLA-DR^−^CD33^+^ cells in colorectal cancer correlating with the nodal and/or distant metastasis, which was considered to be potential MDSCs in this type of cancer although their immunosuppressive function has not been determined in this study 31]. We also evaluated the presence of circulating HLA-DR^−^CD33^+^ cells (Figure S3) and found the similar results with the elevated percentage of this population in colorectal patients (mean 0.860% vs 2.659% for HD vs cancer; p<0.0001) correlating with the tumor stage. Distinct from Lin^−/low^HLA-DR^−^CD11b^+^CD33^+^ MDSCs, this population partially expresses CD11b, CD15 or CD14 31,32], which may represent the mixed MDSC consisting of granulocytic and monocytic cells; however, further investigation is needed to confirm their immunosuppressive function.

Our results are consistent with the change of MDSCs seen in experimental animal tumor models and human cancer showing levels of MDSCs correlate linearly with tumor burden 27,33,34]. When dividing stage IV patients into cased with limited (IV-A stage) or extensive metastasis (IV-B stage), we found that patients with extensive metastasis had the significantly elevated number of circulating MDSCs although the increase in the percentage of MDSCs did not reach the significant level. Intriguingly, we did not observed the significant correlation between MDSCs levels and primary tumor size or serum concentrations of cancer biomarker CEA and CA199. Thus, close correlation between expansion of MDSCs and tumor metastasis indicates MDSCs may play a role in tumor invasion and metastasis 31], and then consequently influence the clinical outcomes of cancer patient 15]. Possible scenario is that factors secreted from tumor cells stimulate the expansion and accumulation of MDSCs in tumor microenvironment and peripheral blood, and recruited MDSCs in turn produce pro-inflammatory and pro-angiogenic factors and promote the proliferation, survival and invasion of tumor cells 35]; therefore, disruption of this vicious circle would hold great promise for enhancing treatment efficacy for colorectal cancer.

Similar to peripheral blood, we also found the markedly elevated level of MDSCs in tumor tissues compared with the surrounding non-cancerous tissues. Previous studies have shown the increased presence of MDSCs defined by different surface markers in the microenvironment of different cancer types 25,31,36,37], including one recent paper documenting the increased percentage of HLA-DR^−^CD33^+^ MDSCs in colorectal cancer correlating with tumor stage and distant metastasis 31]. It should be noted that the description of method for enumerating the percentage of tumor-infiltrating MDSCs was ambiguous and no suitable controls were included in this previous report although they showed the elevated level of MDSCs compared with healthy donors 31]; thus our results unequivocally reaffirmed the presence of increased tumor-infiltrating MDSCs in the colorectal cancer tissues. However, the function of tumor-infiltrating MDSCs is less well investigated. Recently, Corzo and co-workers isolated CD11b^+^CD33^+^CD14^−^ MDSCs from peripheral blood and tissues of head and neck cancer and found that only tumor-resident MDSCs displayed significantly inhibitory effects on PHA-induced T cells proliferation 37], suggesting MDSCs of tumor sites and peripheral lymphoid organs may have distinct function, which indicates that tumor-resident MDSCs might have a more direct clinical relevance and warrants further exploration.

The Lin^−/low^HLA-DR^−^CD11b^+^CD33^+^ circulating MDSCs display the phenotype with high expression of typical myeloid marker CD13, low expression of CD124, CD115 and CD117, devoid of monocytic marker CD14 (Lin^−^) and granulocytic marker CD15 and CD66b and therefore cannot be categorized into monocytic (CD33^+^CD14^+^HLA^−^DR^−/low^) or granulocytic (CD14^−^CD15^+^CD11b^+^CD66b^+^) MDSCs described above. This phenotype is basically similar to the recently identified bone marrow-derived MDSCs (BM-MDSCs) with the promyelocyte-like morphology 15] and C/EBP transcription factor-dependent immunregulatory activity 13]. It has been suggested this population represents immature precursor myeloid cells, which may further differentiate to either monocytic MDSCs or granulocytic MDSCs 38]. Intriguingly, this population expresses CD39 but lacks CD73, two ectonucleotidase molecules mediating conversion of immunostimulatory ATP into immunosuppressive adenosine 39] and also expressed on the surface of human regulatory T cells 40]. CD39 hydrolyses extracellular adenosine tri- and diphosphate (ATP/ADP) to adenosine monophosphate (AMP), which is in turn dephosphorylated by CD73. The resulting adenosine acts to suppress, among others, Th1, Th2, CTL, and NK cells 39,41]. Therefore, combined with CD73 expressed on the conventional T cells, it is tempting to speculate that CD39 may play a role in mediating the suppressive activity of MDSCs on T cells. In fact, recent study shows that CD39 and CD73 are expressed by mouse MDSCs and promote the expansion of and facilitate the suppressive activity of mouse MDSCs 42]. The role of CD39 in the function of human MDSCs is now under active investigation in our lab. In addition, we also detected the slight expression of PD-L1 but not PD-L2 and their receptor PD-1, members of B7 family that are able to regulate immune responses and to induce immunologic tolerance 43]. The role of low PD-L1 expression in human MDSC is unknown although the same expression in the mouse counterpart has been shown to have little effect on the immunosuppressive activity of mouse MDSC 16].

Consistent with previous reports, we found that the circulating MDSCs were able to inhibit T cell proliferation *in vitro*, indicating MDSCs are an important mechanism of cancer-related T cell immunosuppression. In addition, proliferation of T cells in cancer patients was a little bit lower than those isolated from healthy donor even in the absence of MDSCs. The mechanisms underlying Lin^−/low^HLA-DR^−^CD11b^+^CD33^+^ MDSCs-mediated immunosuppression are poorly understood. Samantha S. et al. found that phenotypically similar BM-MDSCs inhibit lymphocyte proliferation by decreasing expression CD3ζ and CD3ε chains on T cells, which may impair the TCR signaling and contribute to immune cell dysfunction 15]. BM-MDSCs also express arginase I, the enzyme capable of depleting the extracellular essential amino acid L-arginine with resultant downregulation of CD3ζ chain and diminished T-cell proliferation 20,44]; however, the role of arginase I in this population needs to be further studied. Recent study shows that CD14^+^HLA^−^DR^−/low^ or CD14^−^CD15^+^CD11b^+^ MDSCs were negatively associated with overall survival in the retrospective analysis of renal cancer patients 45]; therefore, more works are needed to elucidate the clinical relevance of Lin^−/low^HLA-DR^−^CD11b^+^CD33^+^ MDSCs in colorectal cancer, which may lay a theoretical foundation for MDSC-targeting therapies in this type of cancer.

In summary, the data obtained in this study shed a new light on the presence, phenotype and function of MDSCs in patients with colorectal cancer. Further efforts to understand the molecular mechanisms of MDSCs-mediated immunosuppression could potentially lead to novel treatments that circumvent their immunosuppressive effects, providing patient better outcomes.

## Supporting Information

Figure S1 **Correlation analysis between primary tumor size and the percentage and absolute number of MDSCs.** The absolute number (upper graph) and percentage (lower graph) of MDSCs from 18 patients were analyzed to correlate with the primary tumor size by unparametric spearman correlation analysis using Graphpad software.
(TIF)

Figure S2 **Correlation analysis between serum concentration of biomarker CEA and CA199 and the percentage and absolute number of MDSCs.** The absolute number (upper panels) and percentage (lower panels) of MDSCs from all patients (64 cases) were analyzed to correlate with the serum concentration (before treatment) of cancer biomarker CEA (left panels) and CA199 (right panels) by unparametric spearman correlation analysis using Graphpad software.
(TIF)

Figure S3 **The percentage of circulating HLA-DR-CD33+ cells in patients with colorectal cancer.** Fresh whole blood was incubated with a combined anti-Lin, HLA DR, CD33 and CD11b monoclonal antibodies. Acquired cells were analyzed the presence of HLA-DR-CD33+ cells calculated as percentage of peripheral blood mononuclear cells (lymphocyte and monocyte) gated by FS/SS profile.
(TIF)
