# Supplementary figures and images for: Circulating and Tumor-Infiltrating Myeloid-Derived Suppressor Cells in Patients with Colorectal Carcinoma
Source: PLoS One. 2013 Feb 20;8(2):e57114. doi: 10.1371/journal.pone.0057114 (PMC3577767; doi:10.1371/journal.pone.0057114)

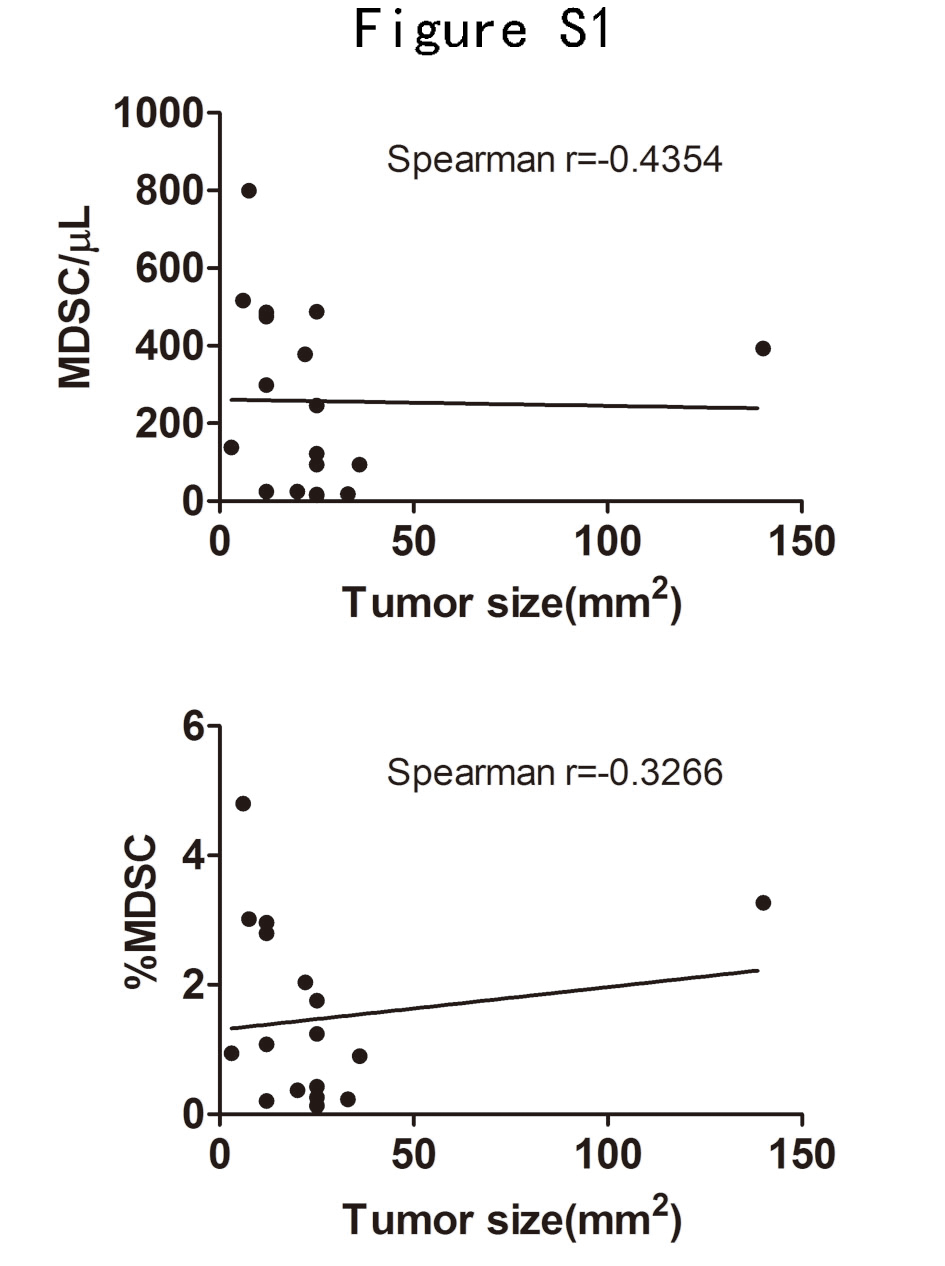

Supplement: Figure S1 — Correlation analysis between primary tumor size and the percentage and absolute number of MDSCs. The absolute number (upper graph) and percentage (lower graph) of MDSCs from 18 patients were analyzed to correlate with the primary tumor size by unparametric spearman correlation analysis using Graphpad software. (TIF) [file pone.0057114.s001.tif]

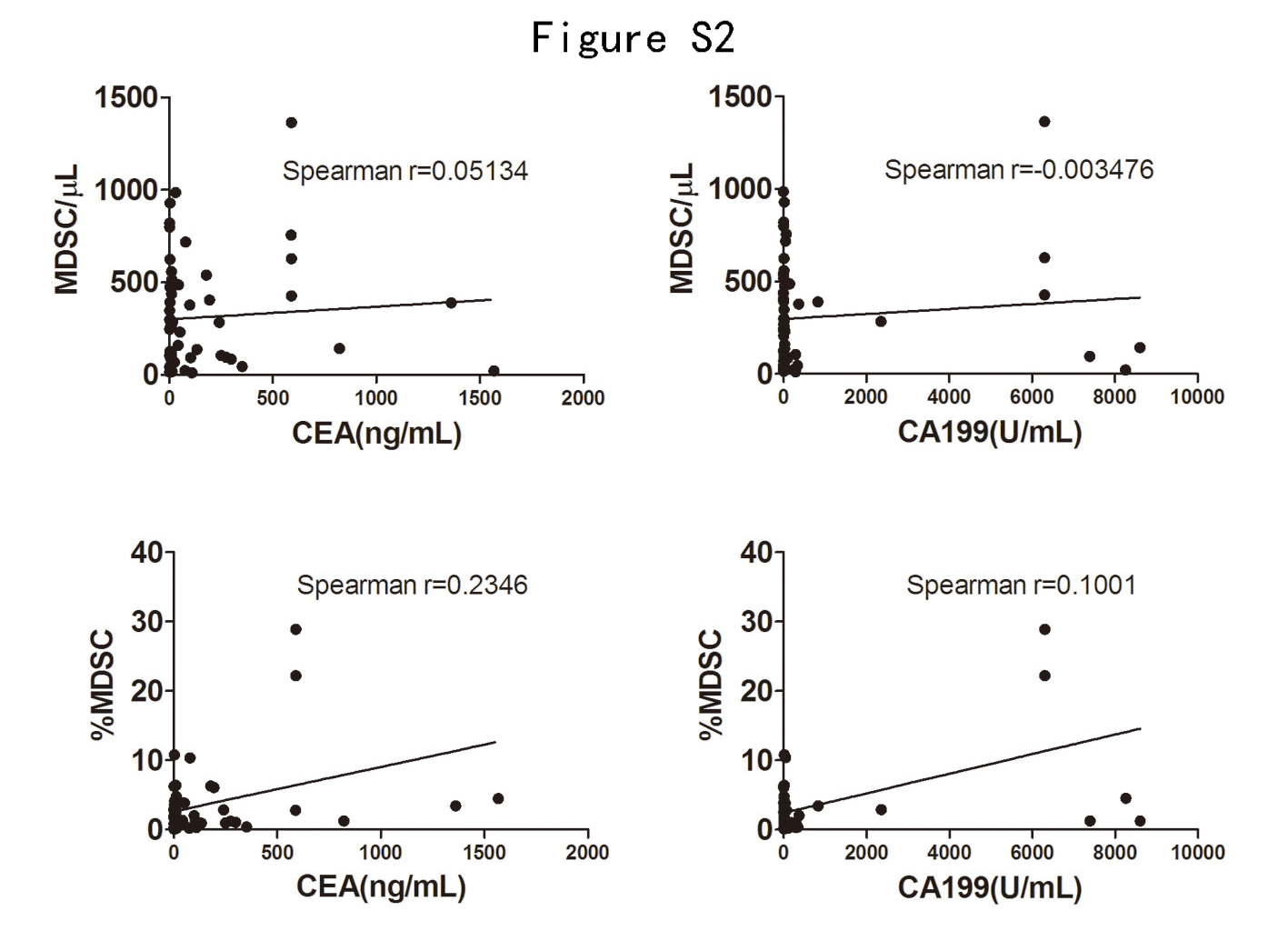

Supplement: Figure S2 — Correlation analysis between serum concentration of biomarker CEA and CA199 and the percentage and absolute number of MDSCs. The absolute number (upper panels) and percentage (lower panels) of MDSCs from all patients (64 cases) were analyzed to correlate with the serum concentration (before treatment) of cancer biomarker CEA (left panels) and CA199 (right panels) by unparametric spearman correlation analysis using Graphpad software. (TIF) [file pone.0057114.s002.tif]

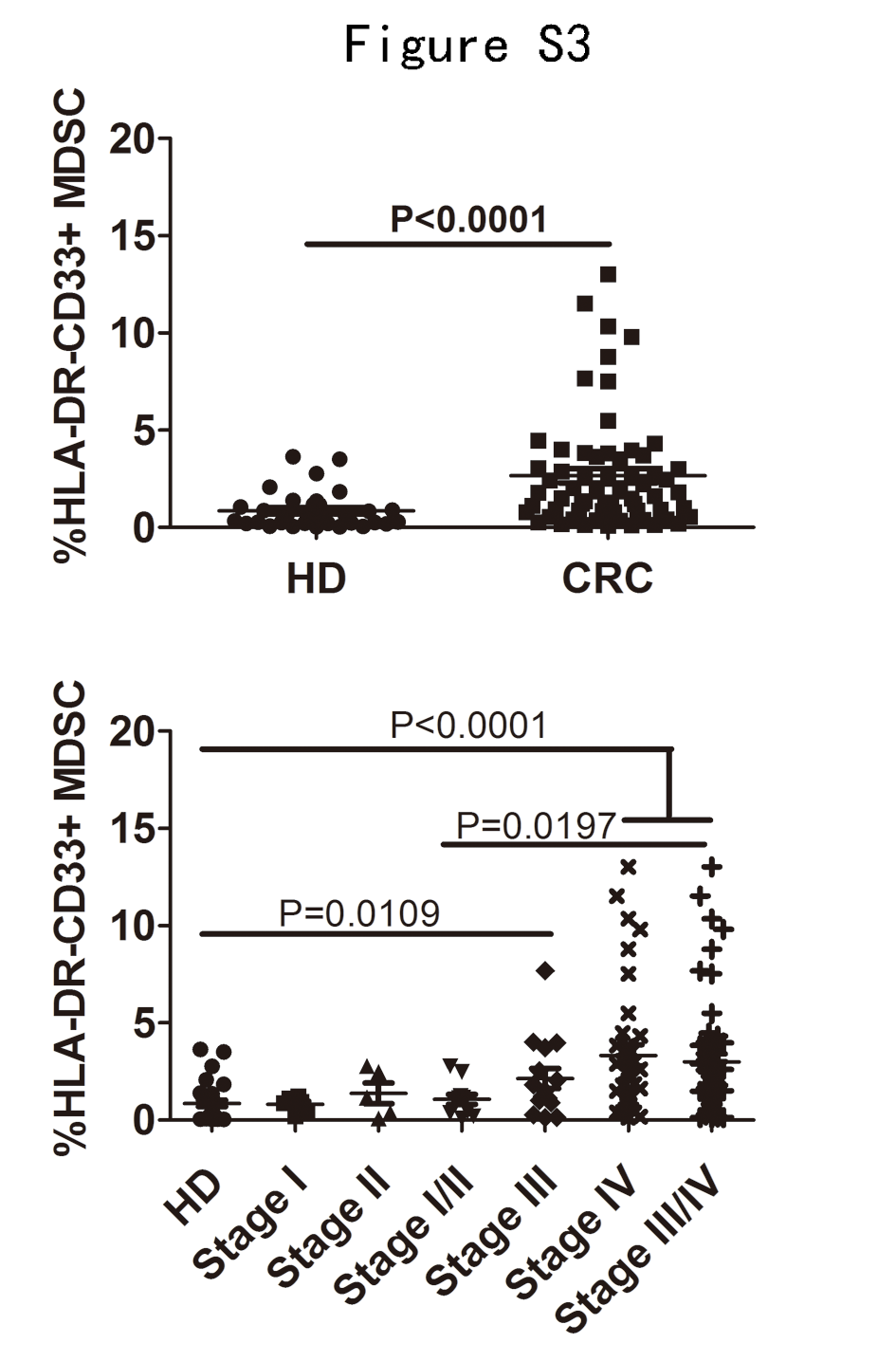

Supplement: Figure S3 — The percentage of circulating HLA-DR-CD33+ cells in patients with colorectal cancer. Fresh whole blood was incubated with a combined anti-Lin, HLA DR, CD33 and CD11b monoclonal antibodies. Acquired cells were analyzed the presence of HLA-DR-CD33+ cells calculated as percentage of peripheral blood mononuclear cells (lymphocyte and monocyte) gated by FS/SS profile. (TIF) [file pone.0057114.s003.tif]
